# Static Electricity—High Frequency Currents

**Published:** 1913-05-03

**Authors:** Alfred C. Norman

**Affiliations:** House Surgeon at Durham County Eye Infirmary.


### ELECTRICITY IN MODERN MEDICINE.

### XXVIII. Static Electricity (*Concluded*)—High Frequency Currents.*

By ALFRED C. NORMAN, M.D. Edin., House Surgeon at Durham County Eye Infirmary.

Static electricity is undoubtedly of great value in the treatment of certain general and functional nervous disorders, especially neurasthenia, but for some reason or other its application is much more common in America than in this country. Probably it is that the climate of America is better suited to the working of the machines.

The following are the more common methods of applying static electricity:—

1. *The Static Bath.*—The patient is seated in a chair on a wood platform that is perfectly insulated from earth by means of four glass legs about 1 foot high. The patient's feet rest upon a flat metal plate on the top of the platform, and this plate is connected with the positive prime conductor of the static machine by a length of flexible metal chain. The negative prime conductor is generally connected to earth by means of another chain and a suitable gas or water pipe. While the machine is working the patient is charged to his full capacity with positive static electricity, the total quantity of electricity he will "hold" depending upon his size—*i.e.*, the surface area of his body—and upon the voltage of the charging current. The earth containing an illimitable quantity of electricity, we may regard the static machine as a sort of pump which is forcing electricity from earth into the patient's body.

The above method of treatment is termed "simple charging" or "the static bath" because the patient's body is bathed in static electricity. The patient's hairs, all being charged with electricity of one polarity, repel one another until they literally stand on end, giving rise to a curious sensation which has been described as that of cobwebs touching the skin. The electricity is of high voltage, consequently it is only awaiting an opportunity to get to earth by the path of least resistance. A certain quantity (depending upon the moisture present) leaks into the air and so reaches earth *via* surrounding objects, and if the operator brings his finger or a suitable electrode near enough to the patient's body a spark will jump across a considerable air-gap, giving rise to a painful sensation to both patient and operator. Most people find the static bath soothing and refreshing; it does not cause "shocks" or pain of any kind, and it is well borne by the most nervous of patients.

2. *The Static Breeze.*—This method is used in conjunction with No. 1 when it is desired to concentrate the current on some particular part of the body. The patient on the insulated platform is connected with the positive conductor of the machine as described under No. 1, the negative conductor being connected to earth. A multiple-pointed metal electrode (connected to earth by means of a brass chain) is placed close to the part of the patient to be treated, and the patient at once feels as if a wind or *breeze* were playing upon the part—hence the term "static breeze." This sensation is due to a silent discharge of electricity passing from the patient through the intervening air to the electrode and thence to earth. The strength of the breeze and its local effects may be varied by varying the distance between the patient and the electrode, but great care must be exercised not to bring the latter close enough to produce sparks. The static breeze is particularly useful in neurasthenia; the electrode, shaped like a crown with many projecting points, is suspended over the patient's head, causing his hair to stand right upon end.

3. *Spark Treatment.*—This is a more drastic method of making local applications of static electricity. While the patient is being charged on the insulated platform, as described under No. 1, the operator passes a metallic ball electrode, connected to earth by a metal chain, so close to the patient that a spark jumps from him to the electrode. This method gives much relief in acute muscular pains and frequently in the pains of locomotor ataxia.

4. *Static Wave Currents.*—This is a modification of the static bath introduced by Dr. Morton, of New York. The patient is connected up exactly as for the static bath, the negative prime conductor of the machine being earthed. The two prime conductors of the machine are then brought so close together that a stream of sparks passes between them, the result being that the positive conductor and the patient are both rapidly charged and discharged while the machine is working. The strength of the current is increased by separating the knobs more widely.

A current somewhat similar to the static wave current could be obtained by connecting a patient, well insulated from earth of course, to one pillar of an *x*-ray induction coil, the discharge from which has been rendered as nearly unidirectional as possible by means of valve tubes, etc. A firm of instrument makers will shortly put on the market an outfit for obtaining these effects from an induction coil.

It might be asked: Why is it the custom to connect one prime conductor of the static machine to earth and then to earth the various electrodes used in giving the breeze or spark treatment instead of connecting the electrodes directly to the conductor in question? There are two reasons for this custom. Firstly, the machine becomes much more powerful when one set of plates is earthed, because their inductive influence is increased as a result of their being connected with an inexhaustible source of electricity. Secondly, the operator is protected against shocks, for he remains at the same electrical pressure as the electrodes he

* Previous articles appeared on Nov. 11, 25; Dec. 9, 30, 1911; Jan. 13, 27, Feb. 17, March 9, 30, April 20, May 4, 25, June 8, July 6, Aug. 3, 17, 31, Sept. 28, Oct. 12, 26, Nov. 9, 30, Dec. 14, 1912; Jan. 11, Feb. 1, March 1, and April 12.

handles—*i.e.*, earth pressure or "zero"—consequently he cannot himself receive any current from them.

Space does not permit of my going into further modifications of static treatment, such as the use of Leyden jars, vacuum electrodes, etc.

### HIGH FREQUENCY CURRENTS.

*High Frequency Electricity* is electricity in a state of extremely rapid oscillation.

When a discharge takes place from a condenser, such as a Leyden jar, a state of electrostatic equilibrium is not reached by a single transference of electricity, but by a number of oscillations to and fro in the discharging circuit that reaches at least 500,000 per second. By means of suitable instruments, known as high frequency transformers, these oscillations can be raised to considerably over one million per second, and such currents when applied to the human body act quite differently from ordinary galvanic or faradic currents. They produce no motor or sensory effects even when currents as large as 500 milliamperes are applied, probably because the oscillations follow each other so rapidly that each one is neutralised by a succeeding one in an opposite direction before it has had time to produce any chemical (ionic) change in the tissues.

High frequency currents have been used in the treatment of almost every disease (except, perhaps, sea-sickness) that flesh is heir to, and the extravagant claims of some of its advocates seemed likely at one time to relegate the whole method to the limbo of quackery. Thanks, however, to the researches of certain workers, we are now in a better position to understand the mode of action of these currents, and, considerably modified and called by another name—"diathermy"—high frequency currents are assured of a definite and important future in electro-therapeutics.

Space does not permit of an elaborate description of apparatus, but it will be necessary to outline the methods used in ordinary high frequency treatment in order that a better understanding may be obtained of the more modern "diathermy."

The apparatus most frequently used for producing ordinary high frequency currents is the D'Arsonval transformer. This consists of two Leyden jars, the inner coats of which are connected with the discharging pillars of an induction coil (or some other source of high voltage electricity) and also with opposite sides of a short spark-gap. The outer coats of the Leyden jars are connected to the ends of a spiral thick copper wire termed a "solenoid." The inner coats of the jars are charged by the induction coil to a high potential and discharge themselves across the spark gap, the result being that a series of charges are induced on the outer coats, and these, discharging through the solenoid, set up oscillations at the enormous rate of one million or more per second. The actual (ohmic) resistance of the copper solenoid is only a small fraction of an ohm, but owing to the great self-induction (see section on induction coils) set up by the rapid oscillations the *inductive* resistance of the wire is enormous; consequently, when a patient is connected with the two ends of the solenoid, the current traverses his body rather than the copper wire, although the resistance of his body in ohms is much greater than that of the wire solenoid.

There are three methods of applying the current to the patient. The first, known as direct application, consists in connecting him directly to the solenoid by means of suitable connecting wires and electrodes, the strength of the current being regulated by varying the number of turns of the solenoid between the ends of the connecting wires. The second method, known as auto-condensation, consists in connecting the patient to one end of the solenoid and a large sheet of metal to the other end, the patient lying on a couch, with the metal sheet fastened beneath the couch. The patient thus forms one armature of a large condenser, the sheet of metal the other armature, and the couch the di-electric. The third method is called auto-conduction, the patient being placed inside, but not touching, a large solenoid. The second is by far the most common method of application.

Local brush discharges or sparks can also be given if an additional piece of apparatus, known as the Oudin resonator, be used. This consists of an ascending spiral of copper wire, the upper end of which terminates in a free metal knob, while the lower part is connected at two points with the solenoid of the D'Arsonval transformer. When the resonator is properly tuned there is a very high degree of self-induction in the upper spirals and a powerful brush discharge or "effluve" is given off from the knob at the top. This discharge may be applied to the patient by means of vacuum or multiple point electrodes, producing a stimulating effect very similar to that of static sparks or breeze.

### The Effects of High Frequency Applications.

High frequency currents are said to increase metabolism; causing more rapid oxidation, more rapid elimination of waste products, and increased heat production. They reduce congestion and relieve pain; give rise to local vaso-dilatation and in some cases reduce blood-pressure.

It is now recognised that practically all the effects of high frequency currents proper are due to their thermal action. These currents, by virtue of their extremely rapid oscillations, are quite innocuous as far as ordinary electrical effects are concerned, hence very large doses can be given. The heat production, however, is directly proportional to the strength of the current, consequently we have in high frequency electricity a means of raising the internal temperature of the body that is only limited by the power of the apparatus at our disposal. Until quite recently this limitation was the cause of failure in many instances, but the introduction of powerful apparatus for wireless telegraphy has rendered possible the medical science of "diathermy," which consists in raising any part of the body to any desired temperature by means of modified high frequency currents. Diathermy will be considered in the next section.

*(To be continued.)*

